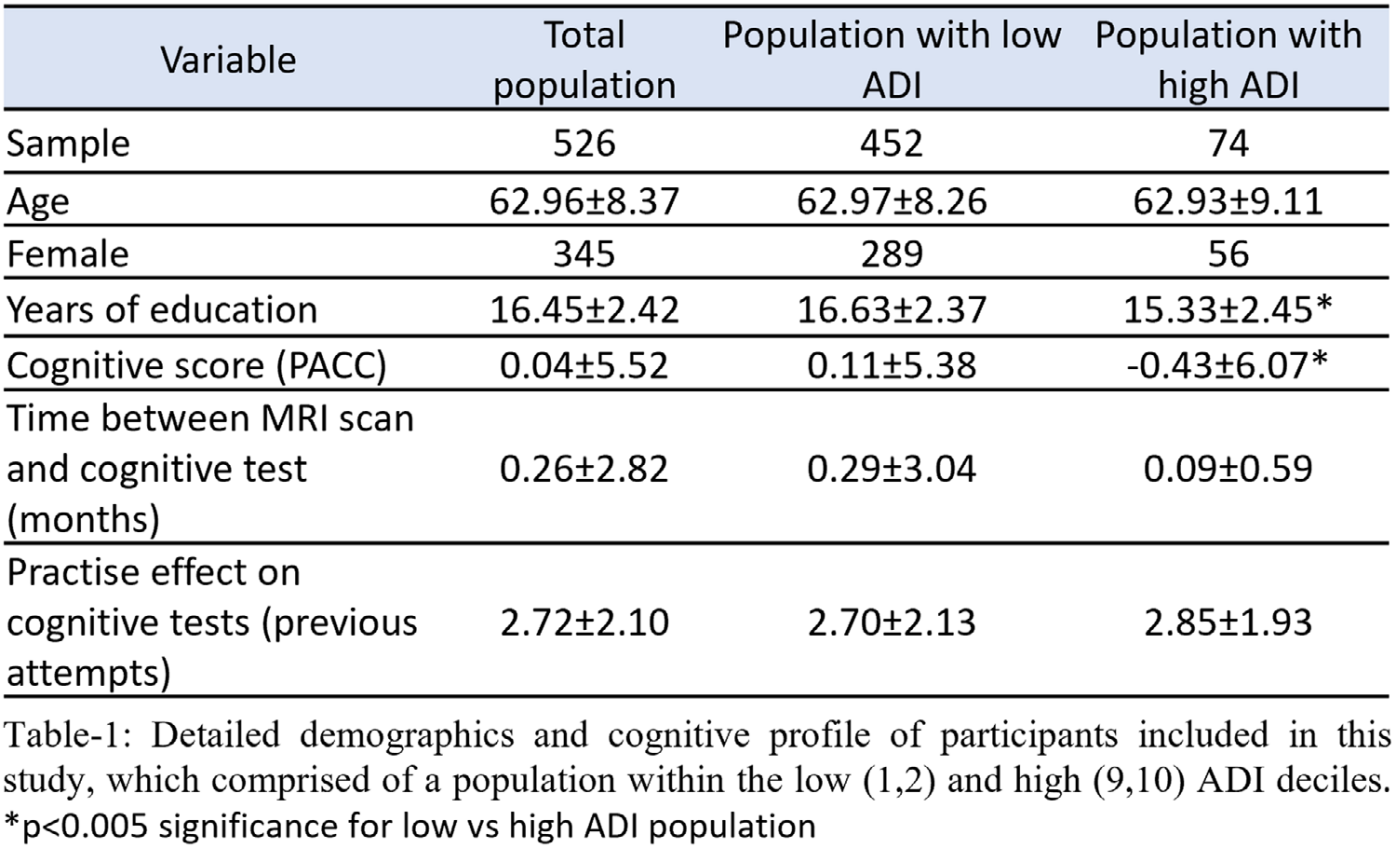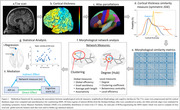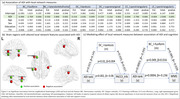# Neighborhood disadvantage is associated with altered cortical organization

**DOI:** 10.1002/alz.093983

**Published:** 2025-01-09

**Authors:** Apoorva Safai, Sterling C. Johnson, Erin M. Jonaitis, Rebecca E. Langhough, W. Ryan Powell, William R. Buckingham, Barbara B. Bendlin, Amy J.H. Kind, Pallavi Tiwari

**Affiliations:** ^1^ University of Wisconsin, Madison, WI USA; ^2^ University of Wisconsin‐Madison, Madison, WI USA

## Abstract

**Background:**

Neighborhood disadvantage is associated with worse health and cognitive outcomes. Morphological similarity networks (MSN) is a promising approach to elucidate cortical network patterns underlying complex cognitive functions. We hypothesized that MSNs can capture intricate changes in cortical patterns related to neighborhood disadvantage and cognitive function, potentially explaining risk for later life cognitive decline among individuals who live in disadvantaged contexts.

**Method:**

This study included cognitively unimpaired participants from the Wisconsin Alzheimer’s Disease Research Center and Wisconsin Registry for Alzheimer’s Prevention. and their neighborhood disadvantage was indexed by the area deprivation index (ADI), a metric representing income, education, employment, and housing quality at the census block group geographic level(n=526, age=62.96±8.377, gender(M:F)=181:345, ADI(L:H)=452,74). We compared MSNs by ADI state rank scores in the two highest (9,10) and lowest (1,2) ADI deciles based on previous studies reporting strongest health effects among individuals living at the highest levels of disadvantage. Cognitive performance was indexed by six tests evaluating memory, executive functioning, and the pre‐clinical Alzheimer’s cognitive composite(PACC). T1‐weighted MRI scans of all participants underwent standard preprocessing steps in CAT12 toolbox MSN was constructed for each participant (Figure‐1). We used linear regression to examine ADI associations with cognitive scores and four local and four global MSN features. Mediating effect of MSN features on the relationship between ADI and cognitive performance was statistically assessed using a non‐parametric bootstrapping (10,000) approach. Model covariates included age, gender, education (years), and total intracranial volume.

**Result:**

ADI rank showed negative association with category fluency(CF)(ß=‐0.176, p=0.039), WAIS‐DigitSymbol(ß=‐0.442, p=0.011), Wechler’s Memory scale(WMS‐LM)(ß=‐0.218, p=0.001) and PACC scores(ß=‐0.056, p=0.001), indicating worse cognitive function among those living in more disadvantaged neighborhoods. Local network features of frontal and temporal brain regions (Figure‐2a, b) differed by ADI group. Centrality of left fusiform and bankssts showed a mediating effect between association of neighborhood disadvantage and performance on the WMS and PACC scores respectively (Figure‐2c).

**Conclusion:**

Our findings suggest differences in local cortical organization by neighborhood disadvantage, which also mediated the relationship between ADI and cognitive performance, providing a possible network‐based mechanism, explaining risk for cognitive decline and dementia associated with disadvantaged neighborhoods. Future work will examine the dosage and timing of neighborhood disadvantage on structural organization of the brain.